# Alcohol and life expectancy

**DOI:** 10.1265/ehpm.25-00101

**Published:** 2025-08-06

**Authors:** Ichiro Wakabayashi, Klaus Groschner

**Affiliations:** 1 Department of Preventive Medicine, School of Medicine, Hyogo Medical University, Nishinomiya, Hyogo 663-8501, Japan; 2 Gottfried Schatz Research Center for Cell Signaling, Metabolism and Aging, Medical University of Graz, Neue Stiftingtalstrasse 6/H03, 8010, Graz, Austria

**Keywords:** Alcohol, Cardiovascular disease, Education, Gender, Income, Life expectancy, Occupation, Socioeconomic inequality

## Abstract

The recent three leading risk factors for global disease burden in the world are, in descending order, high blood pressure, tobacco smoking including second-hand smoke, and alcohol use. Alcohol use increases the risk for many acute and chronic health consequences including cancer, road injury and suicide as well as alcohol use disorder. It is known that there is a U- or J-shaped relationship between alcohol consumption and all-cause mortality. The descending leg of the curve showing this relationship is best explained by a decrease in the risk of cardiovascular disease, especially ischemic heart disease, among light-to-moderate alcohol drinkers. However, this relationship carries risks of confounding and selection bias, including the so-called healthy drinker bias. Furthermore, biogenic compounds other than ethanol present in wine may be partially responsible for the beneficial effect, although this also includes several confounding factors such as the drinking patterns associated with wine preference. While some studies suggest that light-to-moderate alcohol consumption may offer cardiovascular benefits, these findings are likely influenced by confounding factors and do not negate the substantial public health burden associated with alcohol use. In fact, from a population health perspective, reducing harmful alcohol consumption remains a critical priority. Social policies aimed at lowering alcohol intake and limiting drinking opportunities can contribute to longer life expectancy by preventing alcohol-related diseases. Unhealthy alcohol use is one of the four major behavioral risk factors—along with smoking, physical inactivity, and poor diet—that accounted for approximately 50% of all deaths and about six years of life expectancy lost between 2001 and 2008. Targeted interventions are particularly important for men and individuals with lower educational attainment, as alcohol-related mortality is higher in men and contributes more to socioeconomic disparities in life expectancy among men than among women. Alcohol consumption is influenced by socioeconomic factors such as education, income, and occupation. While higher socioeconomic status is associated with more frequent drinking, lower status is associated with higher consumption volume. Given that alcohol-related deaths and life expectancy trends vary across countries and over time, public health strategies should be tailored to specific social and temporal contexts.

## 1. Introduction

Alcohol drinking caused about 2.6 million deaths (men, 2.0 million; women, 0.6 million) worldwide in 2019 according to recent WHO’s statistics [1]. There is a wide spectrum of reasons for alcohol-related deaths such as liver diseases, heart diseases, different types of cancers, infectious diseases, mental health disorders, road traffic accidents, and other injuries including falls, drowning, burns, sexual assault, intimate partner violence and suicide. On the other hand, habitual light-to-moderate alcohol consumption is reportedly associated with a lower incidence of ischemic cardiovascular diseases. Alcohol consumption and drinking-related harm are dependent on socioeconomic circumstances including education, income, occupation, employment status and household assets. In addition, there are age-, gender-, time- and geography-based differences in alcohol-related health issues. The total amount of alcohol intake and pattern of drinking (frequency of drinking and episodic dangerous drinking) determine the impact of alcohol on health outcomes. In this review, we introduce recent evidence regarding the associations between alcohol drinking and total-cause mortality and discuss how alcohol drinking influences life expectancy with focus on socioeconomic aspects.

## 2. Alcohol and all-cause mortality

Alcohol use is an important and commonly recognized determinant of human health. It is needless to say that heavy drinking is inevitably associated with an increased mortality. In a meta-analysis study, age- and sex-matched all-cause mortality in alcohol-dependent subjects was 3.45-fold higher than that in the general population [2]. Another meta-analysis study showed that all-cause mortality rates in men and women with alcohol use disorder (AUD) were 3.38- and 4.57-fold higher, respectively, as compared to control groups without AUD. This supports the concept of alcohol consumption as a lifespan-shortening factor in individuals with AUD and suggests that women with AUD have a generally higher mortality risk than do men with AUD [3]. Interestingly, many studies conducted in various countries revealed a U- or J-shaped relationship between alcohol consumption and all-cause mortality, indicating a dose-dependent dual impact of alcohol with both beneficial and harmful effects of ethanol on health. Light-to-moderate drinkers reportedly have a lower mortality than do abstainers and heavy drinkers. The descending leg of this dose-response curve for alcohol-all mortality relationship results from a decreased risk of cardiovascular disease among those with light-to-moderate alcohol consumption as described below. However, according to a systematic analysis of the Global Burden of Disease (GBD) Study 2016 using 694 data sources of alcohol consumption, alcohol use was the seventh leading risk factor accounting for 2.2% and 6.8% of age-standardized female and male deaths, respectively [4]. According to the recent report of GBD on alcohol-attributable population-level risks of 22 health outcomes [5], theoretical minimum risk exposure level (TMREL) and non-drinker equivalence (NDE) of alcohol consumption (standard drink [10 gram of alcohol] per day) vary by age: TMREL and NDE were 0∼0.603 and 0.002∼1.75, respectively, among individuals aged 15–39 years and were 0.114∼1.87 and 0.193∼6.94, respectively, in individuals aged 40 years and older, in whom the burden-weighed relative risk curve was J-shaped for all regions. Thus, stronger interventions particularly towards younger individuals were recommended to reduce alcohol-attributable global health loss, and the nature of the relative risk curve for older individuals is consistent with the findings of previous studies on the relationship between alcohol consumption and total mortality [5]. Moreover, a multi-country cross-sectional study using data from 193 UN member countries showed that life expectancy at birth was negatively associated with adult alcohol consumption [6]. The average age at enrolment in previous cohort studies was over 50 years in meta-analyses and therefore participants had to have survived to that age in order to be included in the cohort studies, while more than one third of deaths caused by alcohol occur among individuals younger than age 50 years [7]. This type of selection bias results in J-shaped curves since cardiovascular disease is a leading cause of death in individuals aged over 80 years, while the three leading causes of deaths in a younger population aged 15–49 years were tuberculosis, road injuries and self-harm [4], which are associated with alcohol.

According to the Zutphen Study in the Netherlands [8], long-term light alcohol intake compared with no alcohol intake was strongly and inversely associated with cerebrovascular mortality (hazard ratio [HR]: 0.43), total cardiovascular mortality (HR: 0.70) and all-cause mortality (HR: 0.75). In a large-scale prospective study in Japan, J-shaped relationships of alcohol intake with total mortality and cerebro- and cardiovascular mortality were observed both in men and women [9]. In addition to ethanol, wine products contain anti-oxidant compounds represented by resveratrol that have been demonstrated as potentially beneficial for cardiovascular health, although there are several possible confounding factors, including age, drinking pattern, diet, reporting bias and subjective health, for the relationship between wine and cardiovascular health [10]. Independent of total alcohol consumption, long-term wine consumption at an average level of less than half a glass per day was strongly and inversely associated with coronary heart disease mortality (HR: 0.61), total cardiovascular mortality (HR: 0.68) and all-cause mortality (HR: 0.73). Moreover, light wine consumption was associated with 5 years longer life expectancy [8]. Thus, alcohol use increases the risk for many acute and chronic health consequences, but a certain pattern of regular light-to-moderate drinking may have beneficial effects on cardiovascular health [11]. Alcohol abstainers had a life expectancy similar to that of low-to-moderate alcohol drinkers when they did not have a history of risk factors for early death including AUD, risky alcohol drinking, ever having smoked tobacco daily and fair to poor health [12].

In conclusion, we need to consider the effects of habitual alcohol drinking on total mortality and cardiovascular mortality separately. When discussing total mortality, there are possibilities of confounding and selection bias regarding the argument of an inverse relationship between moderate alcohol consumption and total mortality, which was reported in numerous previous cohort studies. On the other hand, as supported by multiple previous studies including meta-analysis reports, it is evident that light-to-moderate alcohol drinking has preventive effects on cardiovascular disease, especially ischemic heart disease. Regardless of reports suggesting an inverse association between light-to-moderate alcohol consumption and cardiovascular disease, strict caution is indicated with respect to regular intake of small amounts of alcohol as a strategy to prevent heart disease since there is always a danger of AUD development and everyone has this possibility in future. Moreover, for healthy habitual drinkers, it is not always easy to restrict their daily alcohol intake persistently at a reasonably low level.

## 3. Country-specific and time-dependent contribution of alcohol-related deaths to life expectancy

The trends in alcohol-related deaths have changed over time and differ across countries. Here, we introduce time-dependent changes of alcohol-related mortality in relation to life expectancy in different countries including European (Western, Central and Eastern) countries, Russia, Nordic countries, the U.S., and Japan.

In a study analyzing life expectancy and alcohol-attributable mortality in 24 Western, Central and Eastern European countries, alcohol-attributable age-standardized mortality rate (ASMR) and life expectancy were higher and lower, respectively, in the Central/Eastern European countries than in the Western European countries in the time from 1990 to 2012. Over the same period, potential gains in life expectancy by eliminating alcohol-attributable mortality were higher in the former countries than in the latter countries. The relative contributions of alcohol to the life expectancy gap between Western and Central/Eastern European countries increased between 1990 and 2005 but declined after 2005. Therefore, reduction of excessive alcohol consumption was considered to contribute to life expectancy convergence across Europe [13].

In Russia, there were three periods: 1965–1984, a period of gradual decline of life expectancy; 1984–2003, a period of massive fluctuations of life expectancy; and 2003–2017, a period of improvement of life expectancy. The strongest negative correlation between changes in life expectancy and harmful alcohol consumption was found in 1984–2003. In the period 2003–2017, life expectancy consistently increased and was independent of harmful alcohol consumption [14].

In Finland during the period from 1991 to 1993, 11% of all deaths among men aged 15 years or over were attributable to alcohol, while alcohol-related deaths were less than 2% of all deaths among women. The proportion of deaths attributable to alcohol peaked at ages around 40 years, being about 45% of all deaths among men and about 25% of all deaths among women and declined rapidly thereafter. This trend may be explained by alcohol-associated accidents and cases of violence that are more frequent and prominent at younger ages [15]. In Finland, alcohol- and smoking-attributable deaths reduced life expectancy by about 4.5 years among men. Alcohol-attributable mortality increased and smoking-attributable mortality decreased over the period from 1988 to 2007 [16]. In the period from 1995 to 2007, about 40%–70% of the between-country differences in life expectancy among Nordic countries was attributed to smoking and alcohol use [17].

On the other hand, between 1999 and 2017 in the U.S., the number of alcohol-related deaths per year among people aged 16 years or older more than doubled from 35914 to 72558, and the age-standardized rate increased by 50.9% from 16.9 to 25.5 per 100000. In 2017, 2.6% of the 2.8 million deaths in the U.S. was directly attributable to alcohol. Almost one third of those deaths resulted from alcoholic liver disease and another 18% resulted from overdoses of alcohol alone or in combination with other drugs. Rates of alcohol-related deaths were higher in men than in women [18]. In the 1980s, the rate of increase in life expectancy in the U.S. was not as fast as that in other wealthy countries. In the U.S., life expectancy rose between 1959 and 2016 from 69.9 years to 78.9 years, while it began declining after 2014 due to an increase in mortality from specific causes such as drug overdoses, suicides and organ system diseases among young and middle-aged adults of all racial groups from the 1990s. Deaths from alcoholic liver disease increased between 1999 and 2017 by about 41% and substantially contributed to the recent increase in mortality [19].

In Japan, ASMR (per 100,000 persons) of alcohol-related deaths in men increased from 4.0 in 1995 to 5.2 between 2010 and 2013 and gradually declined to 5.0 in 2016, while ASMR of alcohol-related deaths in women increased steadily from 0.3 in 1995 to 0.8 in 2016. The ratio of alcohol-attributable ASMR in men to that in women decreased from 13.3 in 1995 to 6.3 in 2016. Amounts of alcohol sale and tax rate in the prefectures of Japan showed positive and inverse correlations, respectively, with their higher alcohol-attributable mortality rates [20]. Therefore, in any regions in the world, social promotion of quitting individual excessive alcohol drinking may contribute to prolongation of life expectancy.

In conclusion, life expectancy and its relation to alcohol-related death differ during history and country, reflecting time-dependent changes and development of socioeconomics and lifestyle. In Eastern Europe and Russia, life expectancy increased and contribution of alcohol-related deaths to life expectancy decreased from around 2005, and as a result, the life expectancy gap between Eastern Europe/Russia and Western Europe declined thereafter. On the other hand, in the U.S., life expectancy recently declined due to an increase in deaths by specific causes including alcohol-related deaths among young and middle-aged adults. In both the U.S., Finland and Japan, it was reported that there was a gender difference in the contribution of alcohol-related death to life expectancy, which is explained in more detail in the section below.

## 4. Alcohol and socioeconomic inequalities

Alcohol consumption contributes to socioeconomic inequalities in health outcomes. According to a systemic review of ten studies including more than 400000 adults and more than 30000 deaths, alcohol use explained up to 27% of the socioeconomic inequalities, including inequalities of education, occupation, employment status, income and household assets, in mortality [21]. Quantity and frequency of drinking were influenced by socioeconomic factors including education, income and occupation: a lower socioeconomic status group drank larger quantities, while a higher socioeconomic status group drank more frequently [22]. In a study comparing four high-income countries and three middle-income countries, lower education and living in poverty were associated with heavier drinking in the seven countries, and drinkers in the middle-income countries had a higher probability to be heavy drinkers than drinkers in the high-income countries. Therefore, associations between socioeconomic disadvantage and heavy drinking depend on country-level income [23]. Middle age-cohorts in high-income countries need to be supplemented with cohorts from different social strata, and cohorts from low and lower-middle income countries with different behaviors to include interactions of alcohol use with other risk factors [24]. Thus, drinking behavior and its relation to life expectancy are considerably influenced by socioeconomic status. The impact and significance of specific socioeconomic factors with respect to the relationship between alcohol consumption and life expectancy are in more detail addressed in the following sections.

## 5. Alcohol and income difference in life expectancy

Analysis of life expectancy in 164 countries showed that alcohol consumption was negatively associated with life expectancy for all income groups [25]. Mortality and life expectancy are influenced by socioeconomic factors, among which income is the strongest factor. In 2003–2007, life expectancy differences between the lowest and highest income quintiles were 11.4 years in men and 6.3 years in women. In the absence of alcohol, these differences would have been 7.4 years (35% less) for men and 4.9 years (22% less) for women [21]. In 2007 in Finland, the gaps in life expectancy between the highest and lowest income quintiles were 12.5 years among men and 6.8 years among women, and one of the most significant factors increasing the gap was alcohol-related diseases [26]. During the period from 1995 to 2007 in four major Nordic countries, about 30–55% of income-related differences in life expectancies within countries were attributed to smoking and alcohol use. Compared with smoking, the contribution of alcohol to income-related difference in life expectancy was greater in men and was smaller in women [17]. Thus, as a conclusion, individuals with lower income have shorter life expectancy due to alcohol-related diseases, typically caused by heavy drinking. Of note, the impact of income on life expectancy shortening by alcohol drinking shows a clear gender difference being less significant in women.

## 6. Alcohol and education difference in life expectancy

In a study investigating 20 European populations from 17 different countries, alcohol-related mortality was higher in a lower educational group than in a higher educational group [27]. In a study using the Austrian death registry between 1991 and 1992, the largest educational mortality disparities compared to all-cause mortality differential was observed for alcohol-associated deaths in men [28]. In the Swedish population at ages of 30–74 years during the period from 1991 to 2008, there was an educational gap in life expectancy between high and low education groups. In 2006–2008, age-adjusted all-cause alcohol-related mortalities per 100000 person years were 23 and 69 in men with high and low levels of education, respectively, and 7 and 20 in women with high and low levels of education, respectively. About 17% of the gap was attributed to alcohol-related deaths in men, while less than 8% of the gap was attributed to alcohol-related mortality in women [29]. The reason for the education difference in alcohol-related mortality is complicated since education inequality in alcohol use differ by countries. In some European countries, more highly educated women were most likely to drink heavily, while among more lowly educated men were at a greater risk for heavy drinking, and social inequalities in alcohol use were reported to differ across groups of countries [30]. In a recent study analyzing individuals aged 30 years and older in European countries, alcohol-attributable mortality increased more among the low-educated than among the high-educated in England and Wales (1972∼2017) and Finland (1987∼2007), whereas alcohol-attributable mortality decreased more among the low-educated than among the high educated in Finland (2007 onwards) and Turin (1972∼2017) [31]. Thus, education inequalities in alcohol-attributable mortality differ by time and country. Individuals with a low level of education may be more susceptible to harmful effects of alcohol due to a tendency for worse healthcare. In general, shortening of life span by alcohol is more often observed in individuals with lower education, but it is difficult to draw conclusions about the significance of educational inequalities in alcohol-related mortality because education influences various aspects of health-related issues.

## 7. Alcohol and gender difference in life expectancy

As afore-mentioned, alcohol-related deaths were more frequent in men than in women [18], while all-cause mortality of individuals with AUD was higher in women than in men [2], possibly due to higher susceptibility to alcohol in women than in men [32]. Vulnerability to the effects of alcohol in women are mainly explained by gender difference in pharmacokinetics of alcohol: Due to their lower body water content and generally smaller size, women absorb alcohol more than men and their blood alcohol concentration is higher than men after consuming the same amount of alcohol. In addition, activity of alcohol dehydrogenase in the stomach is lower in women than in men, which causes a greater portion of ingested alcohol to directly reach and thereby directly expose various organ systems [33]. In Finland during the period from 1991 to 1993, the difference between men and women in life expectancy at the age of 15 years was 7.6 years, about 22% of which was estimated to be attributable to alcohol [15]. In Thailand in 2004–2019, the mortality rate attributable to all alcohol-related causes in a population aged ≥15 years in 2004 was about 4-fold higher in men than in women [34]. In Nordic countries, alcohol had a larger contribution to income-related differences in life expectancies among men than among women [16, 17]. Similarly, the contribution of alcohol-related deaths to the educational gap in life expectancy was higher in men than in women [17].

Gender differences in life expectancy have been traditionally large in central and eastern Europe. The contribution of gender differences in excessive alcohol consumption to the gender gap in life expectancy is substantial: alcohol contributed to at least 15% of the gender difference in mortality in 2012. The absolute contribution of alcohol to the gender gap in life expectancy was strongly correlated with the overall gender differences in life expectancy [35]. Hence, as expected, alcohol-related mortality is higher in men than in women. Therefore, it appears reasonable to conclude that alcohol consumption considerably contributes to the gender gap in life expectancy.

## 8. Alcohol-related mortality by occupation

There has been limited information on the relationship between occupation and life expectancy. It is reasonable to assume that individuals, who are engaged in alcohol-related works, are prone to have alcohol-associated problems including AUD. In a study using large national data in England and Wales in the period of 1991–2000, the highest mortality from alcohol-related diseases and injuries was observed in publicans and bar staff in both sexes and in caterers, cooks, kitchen porters and seafarers in men [36]. In England and Wales in the period of 2001–2005, alcohol-related mortality was highest in people working in the drinks industry including publicans and bar staff [37]. In a cross-sectional study using a large database of the UK biobank, the risk of heavy alcohol consumption was highest in publicans and managers of licensed premises, followed by industrial cleaning process workers and plasterers [38]. In a population-based household survey in the US, the prevalence of AUD was high in laborers in construction and transportation (heavy truck drivers and material movers) independent of demographic variations [39]. In a Swedish register-based study, individuals in manual occupations showed higher risks of AUD and alcohol-related mortality than did individuals in nonmanual occupations [40]. In a study using large-scaled data on hospital admissions in Finland, increased risk of severe alcohol-induced health outcomes was observed mostly in manual workers in craft work, construction and services [41]. Thus, workers with access to alcohol are at high risk of alcohol-related death, and risk of alcohol-induced health problems is higher in workers in manual occupations than in workers in nonmanual occupations. Therefore, promotion programs to effectively reduce alcohol consumption of workers in alcohol-related occupations and manual occupations are necessary to prolong their life expectancy.

## 9. Contribution of alcohol and other unhealthy behaviors to life expectancy

In 2010, the three leading risk factors among 67 risk factors for global disease burden in 21 regions in the world were, in descending order, high blood pressure, tobacco smoking including second-hand smoke, and alcohol use [42]. In Ontario in Canada, people with exposure to all five health risks, smoking, alcohol, food, exercise and stress, had a life expectancy that was more than 20 years shorter than that of people with no health risks. The five risk factors represent a loss of 7.5 years of life expectancy, and smoking, physical activity and inadequate diet had greater impact on life expectancy [43]. Four unhealthy behaviors (smoking, physical inactivity, poor diet, and unhealthy alcohol consumption) were causes of 50% of deaths, equivalent to approximately 6 years of life expectancy lost, in 2001–2008. Smoking was the leading unhealthy behavior contributing to deaths in men, while the burden of excess alcohol was small, which was a reflection of a low prevalence of heavy drinking except at younger ages, at which baseline mortality is low [44]. Individual unhealthy behavioral factors are prone to accumulate, and smoking is a strong confounder for the relation of alcohol with life expectancy. The combined impact of alcohol, smoking and obesity on life expectancy at birth in 2014 compared to that in 1990 declined in men, mainly due to a decline in smoking-attributable mortality, but increased in women due to mortality increases in all three lifestyle-related factors [45]. AUD patients showed significantly shorter telomere lengths than those in a control group, and smoking and diabetes contributed to telomere shortening in AUD patients [46]. Thus, alcohol as well as smoking and diabetes might accelerate aging through telomere shortening, and further molecular medicine-based studies are needed to clarify the mechanism for the effects of alcohol on life expectancy that are independent of alcohol-related diseases.

## 10. Conclusion

**Fig. 1:**
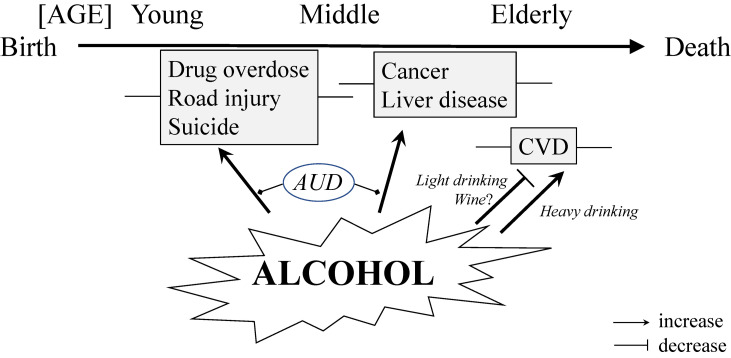
Age-dependent relationships between alcohol and mortality through life. CVD: cardiovascular disease; AUD: alcohol use disorder.

**Fig. 2:**
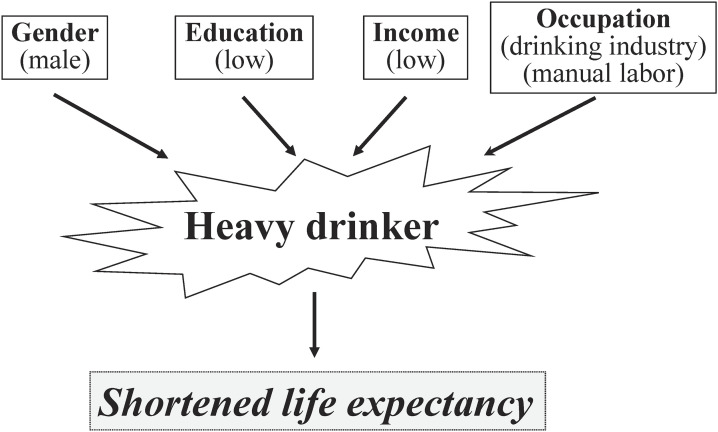
Relationships of gender and socioeconomic factors with life expectancy interacted by alcohol.

Figure 1 summarizes the relation of alcohol with life expectancy. Habitual drinking, especially heavy drinking and binge drinking, increases mortality from alcohol intoxication, road injury and self-harm at a young age and that from cancer and liver disease at middle age and later. On the other hand, habitual light drinking, especially wine intake, decreases mortality from cardiovascular disease at an elderly age. Life expectancy is strongly influenced by inequalities of socioeconomic status including education, income and occupation (Fig. 2). Alcohol consumption considerably contributes to socioeconomic inequalities in health outcomes. The gender difference in life expectancy is also in part explained by alcohol. In addition, there is a gender difference in the contribution of alcohol to socioeconomic inequalities in life expectancy. Alcohol-related deaths are more frequent in men than in women. In general, the contribution of alcohol-related deaths to the socioeconomic gap in life expectancy is higher in men than in women. Modelled interventions for increased alcohol tax, reduced availability of alcohol and a ban on alcohol marketing resulted in a health gain using health-adjusted life years and reduced health inequities between indigenous people and non-indigenous people in New Zealand [47]. Therefore, social policy for reductions in alcohol consumption and opportunity for drinking alcohol is recommended for prolonging life expectancy by prevention of alcohol-related diseases.

## Declarations

### Availability of data and material

No data was used for the research described in the article.
